# Clinical Challenges and Therapeutic Approaches in Managing Deep Carious Lesions Close to the Pulp in Permanent Teeth: A Narrative Review

**DOI:** 10.1155/ijod/5165741

**Published:** 2026-04-18

**Authors:** Negar Gholizadeh, Mahdi Gholamrezaei Saravi, Tahereh Molania, Nafiseh Zarenejad, Hoorieh Abedi

**Affiliations:** ^1^ Department of Restorative Dentistry, Sari Dental School, Mazandaran University of Medical Sciences, Sari, Iran, mazums.ac.ir; ^2^ Oral Medicine Department, Dental Research Center, Faculty of Dentistry, Mazandaran University of Medical Sciences, Sari, Iran, mazums.ac.ir; ^3^ Department of Operative Dentistry, Faculty of Dentistry, Mazandaran University of Medical Sciences, Sari, Iran, mazums.ac.ir

**Keywords:** bioceramics, deep carious lesions, indirect pulp capping, pulp preservation, pulpotomy, regenerative endodontics, review, selective caries removal, vital pulp therapy

## Abstract

**Objectives:**

This narrative review aims to evaluate the clinical challenges and therapeutic approaches for managing deep carious lesions (DCLs) in permanent teeth close to the pulp, with a focus on minimally invasive, pulp‐preserving strategies.

**Materials and Methods:**

A literature review was conducted using PubMed and Google Scholar, with studies searched up to August 2025. Inclusion criteria were clinical trials, systematic reviews, and narrative reviews focusing on DCL management. Emphasis was placed on the biological basis of pulp preservation, diagnostic methods for assessing pulpal health, and the clinical performance of conservative techniques, including selective and stepwise caries removal, indirect and direct pulp capping, and pulpotomy.

**Results:**

The review highlights a paradigm shift from traditional complete caries removal to minimally invasive strategies aimed at preserving pulp vitality. Selective and stepwise caries removal showed success rates up to 97%, while pulpotomy procedures using bioactive materials demonstrated superior biocompatibility and dentin bridge formation. Pulpotomy success rates ranged from 86% to 98% in vital mature teeth, comparable to those of root canal therapy but with lower invasiveness and cost.

**Conclusions:**

Minimally invasive, biologically driven approaches for managing DCLs offer effective pulp vitality preservation and high success rates. The use of bioactive materials, including mineral trioxide aggregate (MTA) and Biodentine, enhances treatment outcomes. These findings are clinically significant, providing evidence‐based guidelines for practitioners seeking to adopt conservative treatment methods for DCLs in vital permanent teeth.

## 1. Background

A deep carious lesion (DCL) is generally understood as a carious lesion that extends into the inner quarter of the dentin, approaching the dental pulp but not yet causing irreversible pulpitis or pulpal exposure [1–3]. Deep lesions are often identified radiographically as caries reaching close to the pulp chamber, and clinically, they may present with minimal symptoms until inflammation of the pulp develops [4]. However, there is currently no universally accepted standard for the precise definition of measurement of DCLs in clinical practice, and further research is needed to standardize these criteria [2, 3].

DCLs are often managed too aggressively, leading to unnecessary root canal treatments (RCTs, endodontic overtreatment) when more conservative, pulp‐preserving options would suffice. Awareness of the latest evidence‐based, minimally invasive techniques such as selective or stepwise caries removal helps clinicians avoid overtreatment and maximize the chances of maintaining a vital, healthy pulp [5, 6]. Knowledge of the challenges, including accurate pulp diagnosis, risk of pulp exposure, and material selection, enables clinicians to tailor their approach, improving the prognosis of the tooth and reducing complications. This is particularly important as the success of treatments like direct pulp capping (DPC) or pulpotomy is closely linked to proper case selection and technique. The management of DCLs has shifted from traditional, invasive methods to more conservative, biologically‐based strategies. Staying informed about these advances ensures that practitioners provide care that aligns with current standards and achieve better long‐term outcomes for patients [6, 7].

There is significant variability in how clinicians manage DCLs, influenced by factors such as academic training, experience, and personal preferences. Understanding the challenges and recommended approaches promotes more consistent, high‐quality care across practitioners and reduces the risk of inappropriate or outdated treatments. Awareness of these issues supports shared decision‐making with patients, allowing clinicians to explain the rationale for conservative management, set realistic expectations, and involve patients in their treatment choices. For dental students and practitioners, knowledge in this area is a marker of up‐to‐date clinical competency and is increasingly emphasized in academic training and continuing education [8–10].

This narrative review focuses primarily on the management of DCLs using minimally invasive, biologically based treatment strategies, including selective caries removal (SCR), vital pulp therapy (VPT), and stepwise excavation. These approaches have gained widespread support due to their potential for preserving pulp vitality and avoiding the need for more invasive treatments such as root canal therapy. While we emphasize minimally invasive procedures, we also acknowledge that traditional complete caries removal remains a valid treatment strategy in certain clinical scenarios, particularly in cases where deep caries are complicated by pulp involvement or other factors that may necessitate more aggressive treatment [11]. We aim to provide a balanced discussion of these approaches, with an emphasis on the emerging trend toward biologically based and minimally invasive strategies.

## 2. Epidemiology and Prevalence

Dental caries is a major global health issue, affecting over 2 billion adults and 520 million children worldwide. In England, 27% of individuals over age 16 have carious teeth, though this figure included all carious lesions, not just DCLs [12]. DCLs extending into the inner dentin and approaching the pulp, often classified as D3 lesions, are less common than shallow or moderate lesions but are of high clinical relevance due to their risk of pulpal involvement. In a recent radiographic study, D3 lesions were the least frequently observed among all carious lesions on approximal surfaces of permanent teeth [13].

In a 2‐year prospective cohort of adults aged 18–64, the baseline prevalence of all active carious lesions (DS1−6) was 83.8%, and for more advanced lesions (DS5−6), which include deep lesions, it was 64.8%. More severe lesions, such as those close to the pulp, were more likely to progress compared to less severe lesions, especially on proximal surfaces and in pits and fissures [14]. First permanent molars are particularly at risk for DCLs, especially in children and adolescents, with prevalence strongly influenced by oral hygiene and dietary habits [15]. DCLs are most frequently found on approximal surfaces and in molars. The presence of adjacent carious surfaces increases the likelihood of deep lesion development [13].

## 3. Pathophysiology and Pulp Response to Caries

Dental caries begins with enamel demineralization, progressing through dentin toward the pulp. As the lesion deepens, bacteria and their byproducts penetrate dentinal tubules, escalating the risk of pulp involvement. The odontoblast layer is the first line of defense, secreting antibacterial compounds and initiating immune responses. However, as caries advances, the odontoblasts may degenerate, compromising this defense [16]. The pulp’s response is primarily inflammatory, with early stages marked by the accumulation of immune cells beneath the affected dentin [17]. Exposure to bacterial products triggers the release of pro‐inflammatory cytokines, including tumor necrosis factor‐alpha (TNF‐α), interleukin 6 (IL‐6), and IL‐8, which mediate inflammation, while anti‐inflammatory cytokines like IL‐10 help modulate the response [18]. As the lesion deepens, immune cell diversity increases, indicating active immune responses, particularly in the coronal pulp beneath DCLs, with potential extension to the radicular pulp [19, 20]. Histological examination reveals cell infiltration and tissue fibrosis, leading to pulp calcifications and, in advanced cases, necrosis. If caries progress unchecked, pulp necrosis can occur, potentially spreading to periapical tissues, with a residual dentin thickness (RDT) ≤1 mm strongly correlating with pulpitis [16, 21].

## 4. Diagnostic Challenges

### 4.1. Clinical Vs. Radiographic Assessment

Clinicians rely on visual and tactile cues, such as lesion depth, color, consistency of dentin, and symptoms reported by the patient. However, these signs are often insufficient to accurately determine the proximity of the lesion to the pulp or the true inflammatory state of the pulp tissue. Overreliance on symptoms and clinical appearance can lead to misjudgment of pulp status and inappropriate treatment choices [22].

Radiographs (periapical, bitewing, or cone‐beam computer tomography [CBCT]) are essential for estimating lesion depth and proximity to the pulp. However, they have limitations: two‐dimensional images can underestimate lesion size, and radiographs cannot reveal the actual histopathological status of the pulp. Advanced imaging like CBCT offers better detail but is not routinely used due to cost and radiation exposure [23]. The most accurate diagnosis requires integrating both clinical and radiographic findings, yet even this approach cannot guarantee precise assessment of pulpal health [22, 23].

### 4.2. Pulp Vitality Tests

Thermal (cold/hot) and electric pulp tests are widely used to infer pulp health based on sensory response. However, they measure nerve response, not true pulp vitality (blood flow), and can yield false positives/negatives, especially in teeth with DCLs, immature apices, or recent trauma. Newer methods such as laser Doppler flowmetry and pulse oximetry attempt to directly measure pulp blood flow, offering a more accurate assessment of true vitality. Despite their promise, these techniques are not yet widely available in clinical practice due to cost, technical complexity, and lack of standardization [24]. In practice, the preoperative vitality test result is considered the most important factor in decision‐making for DCLs, but clinicians must recognize the limitations of these tests and interpret results cautiously [25].

### 4.3. Risk Assessment and Case Selection

Successful management depends on careful case selection, considering factors such as preoperative pulp status, remaining dentin thickness, patient compliance, and accessibility of materials. Teeth with normal pulp vitality and no signs of irreversible pulpitis are preferred candidates for VPT, including SCR or pulp capping. The presence of spontaneous pain, swelling, or radiographic evidence of periapical pathology may indicate irreversible pulpitis or necrosis, requiring more extensive intervention. Limited access to advanced materials and patient factors (e.g., ability to attend multiple visits for stepwise excavation) can influence the choice of treatment and affect outcomes [25, 26].

## 5. Classification and Treatment Thresholds

### 5.1. Shallow Vs. Deep Vs. Extremely Deep Lesions

Shallow lesions are confined to the outer dentin, not approaching the pulp. These lesions have minimal risk of pulpal involvement. Deep lesions extend into the inner third or quarter of the dentin, but with a layer of hard or firm dentin remaining between the lesion and the pulp. These are at risk for pulp exposure during caries removal but may still allow for conservative management [27, 28]. Extremely deep lesions extend very close to the pulp, often with only a thin layer of soft or leathery dentin remaining, or with actual pulp exposure upon caries removal. These lesions present the highest risk for pulpal complications and treatment failure [29].

### 5.2. When Is It “Close to the Pulp”?

A lesion is considered “close to the pulp” when radiographically and clinically, the caries extends to the inner quarter of dentin or when only a thin layer of affected dentin separates the lesion from the pulp chamber [27, 28]. In practice, “close to the pulp” is often determined by the remaining dentin thickness (RDT). An RDT of ≤1 mm is generally considered critical, as it is strongly associated with pulpal inflammation and risk of exposure [27]. Actual pulp exposure may occur during caries removal in extremely deep lesions, distinguishing them from deep lesions where the pulp remains covered [29, 30].

### 5.3. Guidelines and Criteria for Treatment Decision‐Making

The guidelines and criteria for treatment decision‐making are summarized in Table 1. Complete removal of all carious dentin is now considered overtreatment for deep/extremely deep lesions. SCR is preferred to reduce the risk of pulp exposure and preserve vitality [27, 28, 31]. Preoperative pulp vitality testing is the most critical factor in deciding between VPT and RCT [25]. For deep lesions without exposure, glass ionomer or resin‐modified glass ionomer bases are commonly used. For exposures, mineral trioxide aggregate (MTA), Biodentine, or calcium hydroxide (CH) are preferred for DPC or pulpotomy [25, 32, 33]. DPC is favored for exposures <1 mm; larger exposures (>1–2 mm) often lead to RCT, especially if symptoms suggest irreversible pulpitis [30]. Management is subjective and depends on the clinician’s experience, patient factors, and material availability. Consistency in terminology and unified criteria are needed to reduce variability in practice [27, 31].

**Table 1:** Guidelines and criteria for treatment decision‐making in deep carious lesions.

Lesion depth	Clinical approach	Key criterion/guideline
Shallow/moderate	Complete caries removal and restoration	Low risk of pulp exposure; standard restorative approach
Deep	Selective (partial) caries removal, indirect pulp therapy	Leave affected dentin over pulp to avoid exposure; restore with biocompatible material
Extremely deep	Stepwise excavation, DPC, or partial pulpotomy if exposure occurs	If pulp exposed control bleeding, assess pulp status, use biocompatible capping material (e.g., MTA, Biodentine)

Abbreviations: DPC, direct pulp capping; MTA, mineral trioxide aggregate.

## 6. Contemporary Therapeutic Approaches

While minimally invasive approaches like SCR and VPT are gaining traction due to their focus on pulp preservation and less invasiveness, it is important to acknowledge that complete caries removal remains a viable option in specific cases. According to Al‐Ali and Camilleri [11], complete caries removal is often recommended for cases involving deep caries where pulpal health cannot be guaranteed. Their review emphasizes that complete nonselective caries removal offers a more predictable outcome, particularly when the pulp is at risk or when dealing with deep lesions that may be complicated by pulp exposure.

### 6.1. Indirect Pulp Capping

#### 6.1.1. Procedure

The first step in indirect pulp capping is caries removal; initially, all infected dentin is removed from the cavity walls, and then, a thin layer of affected (not infected) dentin is left on the pulpal floor to avoid pulp exposure. The next step is disinfection, that is, cleaning the cavity with a suitable disinfectant, such as chlorhexidine. Subsequently, the chosen pulp capping material is applied over the remaining dentin near the pulp. Finally, the cavity is sealed with a temporary or permanent restorative material. In some protocols, a temporary restoration is placed first, with a permanent restoration after a short interval of 2–3 weeks. Afterwards, the patients are followed up with clinical and radiographical monitoring of symptoms, pulp vitality, and reparative dentin formation at intervals [34–36].

#### 6.1.2. Materials

I. CH: traditional material, induces reparative dentin but may have lower long‐term sealing ability and higher solubility [35, 37].
II. MTA: bioceramic, superior sealing, excellent biocompatibility, promotes dentin bridge formation, and shows higher success rates than CH [35, 37].
III. Biodentine: calcium silicate‐based, bioactive, easy handling, and comparable or superior outcomes to MTA and CH [34, 35].
IV. TheraCal LC: light‐cured, resin‐modified calcium silicate, high dentin formation, and excellent clinical performance [33, 36].
V. Resin‐Modified Glass Ionomer Cement (RMGIC): good biocompatibility and clinical results, used as a liner [33, 38].

#### 6.1.3. Clinical Outcomes and Success Rates

Table 2 shows the 6–12‐month success rates of each material. Regarding long‐term outcomes, a 10‐year retrospective study found indirect pulp capping had a 93.8% success rate, significantly higher than DPC (23.8%) [39]. Considering pain and symptoms, all tested materials have resulted in significant reduction in pain and have maintained pulp vitality, with no significant difference in pain scores between RMGIC and TheraCal LC [38]. In terms of dentin formation, TheraCal LC has shown the highest percentage gain in dentin formation, followed by RMGIC and Dycal (CH) [33].

**Table 2:** Success rates of materials used for indirect pulp capping within 6–12 months.

Material	Success rate (6–12 months)	Key findings
Biodentine	80%–91.7%	High bioactivity, suitable for indirect pulp capping, forms dentin bridge [34, 35]
MTA	83%–96%	Superior to CH, maintains pulp vitality, reduces pain [35, 37]
CH	58%–100%	Traditional, lower‐long‐term success, still effective [35, 37]
TheraCalLC	81%–96.2%	High dentin formation, excellent outcomes [33, 36, 38]
RMGIC	High (comparable to TheraCal LC)	Effective, good clinical results [33, 38]

Abbreviations: CH, calcium hydroxide; MTA, mineral trioxide aggregate; RMGIC, resin‐modified glass ionomer cement.

The comparison of indirect pulp capping and DPC reveals a significant difference in long‐term success rates, with indirect pulp capping achieving 93.8% success over 10 years, while DPC had only a 23.8% success rate [39]. This underscores the importance of long‐term studies to evaluate treatment durability. Among materials, bioceramic materials (MTA, Biodentine, TheraCal LC) consistently outperform CH in maintaining pulp vitality and promoting dentin bridge formation [33, 35, 37]. Additionally, stepwise excavation (97% success rate) outperforms single‐visit indirect pulp capping (82.4%) in preserving pulp vitality, highlighting its potential as a superior method for treating DCLs [40]. Moreover, there was no significant difference in success rates between liners (TheraCal LC, Dycal) and no liner after 1 year, indicating that liners provide comparable short‐term outcomes but require further long‐term validation [36].

### 6.2. Stepwise Excavation

#### 6.2.1. Procedure

Stepwise excavation is a conservative, staged approach for managing DLCs that are close to the pulp, aiming to preserve pulp vitality and minimize the risk of pulp exposure. In the first visit, caries is removed from the cavity walls, while soft dentin over the pulpal floor is left intact to avoid pulp exposure; a biocompatible liner is placed, and the cavity is sealed temporarily. Over an interim period of 2–6 months, this sealed environment promotes arrest of the carious process and formation of reparative dentin. At the second visit, the cavity is reopened, the previously soft dentin—now harder and drier—is reassessed and selectively removed, followed by placement of a final liner and permanent restoration [40–42].

#### 6.2.2. Indications and Rationale

Indications include DLCs in permanent teeth where complete caries removal would likely result in pulp exposure and teeth with vital pulps, no signs of irreversible pulpitis, and no spontaneous pain, especially suitable for young permanent teeth where pulp preservation is critical for continued root development [40, 41]. The rationale comprises pulp preservation, biological repair, and clinical success. By avoiding immediate complete caries removal, the risk of pulp exposure and subsequent need for endodontic treatment is significantly reduced. The sealed environment allows the pulp–dentin complex to respond by forming reparative dentin and reducing bacterial activity. In addition, studies show higher rates of pulp vitality preservation and lower rates of pulp exposure compared to direct, complete excavation [40–42].

#### 6.2.3. One‐Step Vs. Two‐Step Protocols

The description and comparison of the two protocols are shown in Table 3. Both protocols are effective, but the two‐step (stepwise) approach may offer slightly higher success in maintaining pulp vitality, especially in very deep lesions. However, the one‐step protocol is less time‐consuming and avoids the risk of pulp exposure during reentry. Recent studies suggest that, for many cases, a well‐sealed one‐step SCR may be sufficient, with similar long‐term outcomes [43, 44]. Stepwise excavation has demonstrated high success rates (up to 97%) in preserving pulp vitality, significantly outperforming indirect pulp capping in some studies [40]. Moreover, the risk of pulp exposure is much lower with stepwise excavation (17.5%) compared to direct complete excavation (31.5%–40%) [41]. Further, after the interim period, the dentin becomes harder and drier, indicating arrest of the carious process and improved conditions for final restoration [40, 42].

**Table 3:** One‐step vs. two‐step excavation in deep carious lesions.

Protocol	Description	Success/considerations
One‐step	Selective (partial) caries removal in a single visit, leaving affected dentin over the pulp, then permanent restoration.	Lower risk of pulp exposure, avoids need for reentry, relies on good case selection and sealing [43, 44]
Two‐step	Stepwise excavation as described above: initial partial removal, temporary seal, reentry after 2–6 months for final excavation and restoration.	Higher pulp vitality preservation, allows further hardening of dentin, but requires patient compliance for follow‐up [40–42].

Abbreviations: CH, calcium hydroxide; MTA, mineral trioxide aggregate; RMGIC, resin‐modified glass ionomer cement.

### 6.3. SCR

#### 6.3.1. Definitions and Concepts

SCR is a minimally invasive technique for managing DCLs, especially those approaching the pulp in permanent teeth. The core concept is to remove all infected, soft dentin from the cavity periphery (walls and enamel–dentin junction) while intentionally leaving a layer of affected, softer dentin over the pulpal floor. This approach aims to avoid pulp exposure and preserve pulp vitality, in contrast to traditional complete (nonselective) caries removal, which increases the risk of pulpal complications [26, 45]. There are two types of SCR: selective removal to soft dentin (SRSD) and selective removal to firm dentin (SRFD). In SRSD, caries is removed until only soft, demineralized dentin remains over the pulp, while in SRFD, caries is removed until firmer, leathery dentin is left, but not all the way to hard, sound dentin.

#### 6.3.2. Evidence‐Based Advantages

SCR significantly lowers the risk of accidental pulp exposure compared to nonselective (total) removal, which is especially important in deep lesions [26, 45, 46]. Clinical trials show that SRSD achieves higher or comparable success rates in maintaining pulp vitality and tooth survival compared to more aggressive techniques. For example, a 2‐year randomized trial found SRSD had a 100% success rate (with liner) and 93.5% (without liner), both higher than nonselective removal (82.4%) [46]. By avoiding unnecessary removal of affected but remineralizable dentin, SCR preserves more natural tooth tissue, supporting long‐term tooth strength and function (45). The technique is less invasive, often less painful, and reduces the need for more complex treatments like root canal therapy [26, 45]. SCR is associated with fewer postoperative complications, such as pain, abscess, or periapical pathology, and supports the biological healing potential of the pulp–dentin complex [45, 46]. Systematic reviews and meta‐analyses recommend SCR as the treatment of choice for DCLs in vital permanent teeth, especially when the lesion extends beyond two‐thirds of the dentin thickness [26].

### 6.4. DPC and Pulpotomy

#### 6.4.1. When Exposure Occurs

DPC is indicated for small, pinpoint exposures in teeth with a healthy or reversibly inflamed pulp, especially when the exposure is mechanical or due to caries removal and the pulp is not irreversibly inflamed [47, 48]. Meanwhile, pulpotomy is indicated for larger exposures, persistent bleeding after exposure, or when the pulp is more inflamed but still vital. It is also considered when the exposure occurs in teeth with symptoms suggestive of irreversible pulpitis, provided hemostasis can be achieved after removal of the inflamed tissue [49–51].

#### 6.4.2. Partial Vs. Full Pulpotomy

Both techniques are effective for VPT in permanent teeth with DCLs. The choice depends on the extent of inflammation and ability to control bleeding [49, 51]. The extent of removal, indications, success rate, pain control, and clinical decision are compared between partial and full pulpotomy in Table 4.

**Table 4:** Comparison of partial and full pulpotomy for deep carious lesions.

Aspect	Partial pulpotomy (Cvek)	Full/coronal pulpotomy
Extent of removal	2–3 mm of inflamed coronal pulp	Entire coronal pulp to canal orifices
Indication	Small exposures, limited inflammation	Larger exposures, more extensive inflammation
Success rate	Comparable to full pulpotomy; slightly lower in some studies	High, often >85%–90% at 1 year
Pain control	Both provide effective pain relief; full pulpotomy may result in lower pain scores post‐op [49]	
Clinical decision	Start with partial if possible; proceed to full if bleeding persists or inflammation is extensive [49, 51]	

#### 6.4.3. Long‐Term Outcomes and Materials Used

Success rates in DPC vary significantly depending on the material used and follow‐up period. MTA demonstrates strong short‐term (91% at 6 months) and long‐term (81% at 4–5 years) success rates, while Biodentine shows slightly better short‐term success (96% at 6 months) and maintains strong long‐term results (86% at 2–3 years). In contrast, CH, despite being a traditional material, shows considerably lower long‐term success (56% at 4–5 years) due to issues like poor sealing and the potential for tunnel defects [47, 52, 53]. Partial and full pulpotomies show high success rates (86%–98% at 1–2 years), often outperforming DPC in cases of irreversible pulpitis, with some studies reporting success rates exceeding 90% at 18–24 months [49, 50, 54–58]. Notably, MTA remains the gold standard for both DPC and pulpotomy due to its high biocompatibility and sealing ability, with Biodentine showing comparable or slightly better outcomes [47, 49, 52–55]. Newer materials like Endocem MTA Premixed show promise, and advanced platelet‐rich fibrin (A‐PRF+) is still experimental, but with some evidence of dentin bridge formation [54, 59].

**Figure 1:**
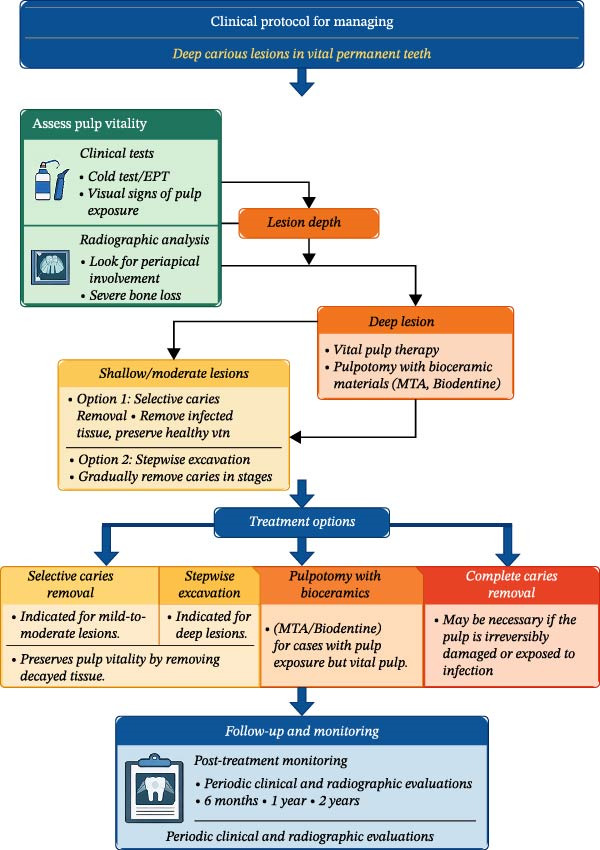
| Clinical decision‐making protocol for managing deep carious lesions in vital permanent teeth. This algorithm outlines a structured, evidence‐based approach for diagnosing and treating deep carious lesions, focusing on treatment options such as selective caries removal, stepwise excavation, and vital pulp therapy.

To guide clinical decision‐making, a detailed algorithm has been developed, which summarizes the steps for assessing pulp vitality, determining lesion depth, and choosing the appropriate treatment based on clinical and radiographic findings (Figure 1).

## 7. Biomaterials in Pulp Preservation

Table 5 compares bioactive materials in terms of success rate, dentin bridge quality, pulpal inflammation, handling, and cost. Considering the antibacterial properties, CH has a strong initial antibacterial effect due to high pH, but this effect diminishes over time [64]. Alternatively, MTA, Biodentine, and bioceramics have moderate antibacterial activity, mainly due to alkaline pH, but less than CA [64, 65]. As for sealing ability, CH has poor long‐term seal and is prone to dissolution and microleakage [64, 65]. Contrarily, MTA has excellent sealing due to expansion on setting and biocompatibility [64, 66]. Biodentine is comparable or superior to MTA, with good marginal adaptation and low solubility [64, 65]. Bioceramics generally have high sealing ability, low solubility, and good adaptation [64, 65]. Regarding the regenerative potential, CH stimulated dentin bridge formation, but bridges are often incomplete and porous [64, 67]. MTA, Biodentine, and bioceramics promote thicker, more continuous dentin bridges with less inflammation and better pulp healing [64, 67–69].

**Table 5:** Comparison of bioactive materials in pulp preservation for deep carious lesions.

Properties	CH	MTA	Biodentine	Bioceramics (e.g., CEM, NeoMTA)
Success rate	48%–58% (6–9 months) [60, 61]	72%–92% (6–12 months) [60–62]	83%–86% (6–12 months) [60, 62]	86%–91% (6–18 months) [60, 63]
Dentin bridge quality	Thin, porous, tunnel defects	Thick, continuous, fewer defects	Thick, continuous	Comparable to MTA, good quality
Pulpal inflammation	More frequent	Minimal	Minimal	Minimal
Handling	Easy, but poor seal	Good, but longer setting time	Good, fast set	Good, improved handling
Cost	Low	Moderate–high	Moderate	Moderate–high

Abbreviations: CEM, calcium‐enriched mixture (cement); CH, calcium hydroxide; MTA, mineral trioxide aggregate.

### 7.1. Clinical Evidence

MTA and Biodentine consistently outperform CH in both direct and indirect pulp capping, with higher success rates and better histological outcomes [35, 60, 62, 63, 67, 70]. Bioceramics (e.g., calcium‐enriched mixture [CEM] cement, NeoMTA) show similar or superior results to MTA, with high rates of pulp vitality preservation and dentin bridge formation [63, 69]. Moreover, MTA and Biodentine maintain high success rates (>80%) at 1–2 years, while CH shows a significant drop in success over time due to dissolution and poor sealing [60, 62, 63, 67]. Meanwhile, bioceramics are emerging as reliable alternatives, with studies showing comparable or better outcomes than MTA [63, 69].

### 7.2. Future Trends

Premixed, fast‐setting, and easier‐to‐handle bioceramics are being developed. Bioactive molecules and growth factors are incorporated to enhance pulp regeneration. The focus of research is on materials with improved antibacterial properties and long‐term stability [61, 64]. Research into stem cell‐based therapies and bioactive scaffolds is ongoing, aiming for true pulp–dentin complex regeneration [61, 64].

## 8. Prognostic Factors and Outcomes

### 8.1. Age, Lesion Depth, and Symptoms

Younger patients (<40 years) have significantly higher success rates for VPT procedures such as DPC and pulpotomy. For example, one study found a success rate of 90.9% in patients under 40, compared to 73.8% in those 40 or older. Age‐related changes in pulp tissue, such as reduced vascularity and cellularity, may contribute to lower healing potential in older adults [71]. Deeper lesions (involving >2/3 of dentin) increase the risk of pulp exposure and inflammation, but selective or stepwise caries removal techniques can help preserve pulp vitality with high success rates. The severity and activity of the lesion (active vs. arrested) also influence the likelihood of progression and treatment outcome. Furthermore, teeth with reversible pulpitis (mild, provoked pain) have better outcomes than those with symptoms of irreversible pulpitis (spontaneous, lingering pain). The presence of spontaneous pain, sensitivity to percussion, or radiographic signs of periapical pathology are negative prognostic indicators and may necessitate more invasive treatment [14, 43, 44].

### 8.2. Operator Experience and Follow‐Up Protocols

Clinical judgment and experience play a crucial role in decision‐making, especially in determining the extent of caries removal and managing pulp exposures. Less experienced practitioners may be more likely to overtreat (e.g., unnecessary endodontic therapy) or be less comfortable with conservative approaches like SCR. Consistent protocols and training can help standardize care and improve outcomes [5, 31].

Regular follow‐up is essential for monitoring pulp vitality, pain, and radiographic changes. Success is typically defined by the absence of symptoms, positive pulp vitality tests, and no radiographic evidence of pathology at 6–18 months. Longer‐term follow‐up (up to 5 years) is recommended, as some failures may occur late due to microleakage or restoration failure [44, 71, 72]. The predictors of success and failure are summarized in Table 6. Additional factors include material choice, patient compliance, and restoration quality. Use of modern bioceramics (MTA, Biodentine) is associated with higher success rates than CH. Adherence to recall visits and oral hygiene instructions supports long‐term success. Also, immediate and durable restoration reduces the risk of microleakage and secondary caries [44, 72].

**Table 6:** Predictors of success and failure in managing deep carious lesions.

Predictor	Associated with success	Associated with failure
Younger age	Higher healing potential	Older age, reduced pulp vitality
Shallow/moderate lesion depth	Lower risk of pulp exposure	Deep lesions, higher risk of exposure
Mild/no symptoms	Reversible pulpitis, better outcomes	Irreversible pulpitis, spontaneous pain
Operator skill	Conservative, evidence‐based approach	Overtreatment, inconsistent technique
Good coronal seal	Prevents microleakage, maintains pulp health	Poor seal, restoration failure
Regular follow‐up	Early detection of complications	Missed late failures

## 9. Current Controversies and Debates

### 9.1. Pulpotomy in Mature Teeth as a Definitive Treatment

The key debate is whether pulpotomy can serve as a permanent alternative to RCT in mature permanent teeth with irreversible pulpitis. In the traditional view, RCT has been the gold standard for mature teeth with irreversible pulpitis, and pulpotomy is reserved for primary or immature permanent teeth. However, recent randomized controlled trials and systematic reviews show that full pulpotomy in mature permanent teeth can achieve clinical and radiographic success rates comparable to RCT (clinical: 81%–98%, radiographic: 38%–95%) [57, 73–75]. Pulpotomy is less invasive, preserves more tooth structure, and is less technically demanding [51, 58]. Success is highly dependent on case selection, operator skill, and use of modern bioactive materials (e.g., MTA, Biodentine) [57, 58, 74]. Additionally, some systematic reviews note insufficient long‐term evidence to universally recommend pulpotomy as a replacement for RCT, but it is increasingly accepted as a viable option in select cases (51, 74).

Nevertheless, long‐term durability and risk of late failure remain concerns. Some clinicians are hesitant to adopt pulpotomy as a definitive treatment due to limited high‐quality, long‐term studies. Guidelines are evolving, with more support for pulpotomy in mature teeth, especially where RCT is not feasible or as an interim measure [51, 57, 58, 74].

### 9.2. Cost‐Effectiveness and Patient Compliance

Pulpotomy is significantly less expensive than RCT, both in direct treatment costs and chair time [73, 76]. Economic models show pulpotomy is highly cost‐effective, especially in settings with limited resources or low willingness‐to‐pay thresholds [76]. As willingness‐to‐pay increases, RCT may become more cost‐effective due to slightly higher long‐term tooth survival, but the difference is often marginal [76]. Furthermore, pulpotomy is less time‐consuming, often completed in a single visit, which improves patient compliance and satisfaction [58, 73, 77]. Lower cost and reduced treatment complexity make pulpotomy more accessible, especially in public health settings or for patients with financial constraints (77). Studies report high patient satisfaction and similar or better pain relief compared to RCT [73, 77, 78].

## 10. Future Perspectives and Research Directions

### 10.1. Regenerative Endodontics and VPT

Regenerative endodontics is rapidly evolving, aiming to restore the natural structure and function of the pulp–dentin complex rather than simply preserving tooth structure with inert materials. This approach leverages advances in tissue engineering, stem cell biology, and biomaterials to promote true regeneration of dental tissues [79–81]. Key innovations include stem cell‐based therapies, biomimetic scaffolds, growth factors and bioactive molecules, and three‐dimensional (3D) bioprinting. Dental pulp and mesenchymal stem cells can be used to regenerate pulp tissue and dentin. Natural and synthetic scaffolds may support cell growth, differentiation, and vascularization within the root canal [82, 83]. Also, signaling molecules can be incorporated to enhance tissue repair and regeneration. Moreover, emerging techniques in 3D bioprinting allow for precise placement of cells and scaffolds, potentially enabling in situ regeneration of pulp tissue [79, 84]. Clinical translation is ongoing, with current protocols showing promise in immature teeth and early‐stage trials in mature teeth. However, challenges remain in achieving predictable, long‐term outcomes and fully functional tissue regeneration [85, 86].

### 10.2. Artificial Intelligence (AI) and Digital Diagnostics

AI is transforming diagnostics and decision‐making in endodontics: AI algorithms can detect carious and periapical lesions, assess root morphology, and predict case difficulty with high accuracy, supporting clinicians in diagnosis and treatment planning [87, 88]. Machine learning models are being developed to forecast treatment outcomes and guide personalized therapy. AI can streamline patient data management, assist in history‐taking, and support real‐time clinical decision‐making. Research is exploring chairside point‐of‐care tests (e.g., rapid C‐reactive protein assays) to objectively assess pulp inflammation, potentially improving diagnostic precision and reducing reliance on subjective symptoms [89]. Future directions include the integration of AI with digital imaging, electronic health records, and intraoperative diagnostics to enable more accurate, standardized, and efficient care [87, 88].

### 10.3. Long‐Term Clinical Trials and Standardization

Despite advances in materials and techniques, there is a recognized gap in high‐quality, long‐term clinical trials comparing different VPT strategies and regenerative approaches [22, 90]. Current reliance on subjective symptoms leads to variability in treatment decisions. There is a pressing need for objective, reproducible diagnostic tools and consensus on case definitions (22, 89). Variations in clinical protocols, operator experience, and follow‐up regimens hinder the ability to compare outcomes across studies and settings. Standardized definitions of success and failure, including both clinical and patient‐reported outcomes, are essential for meaningful comparisons and guideline development.

Research priorities include multicenter, randomized controlled trials with long‐term follow‐up to assess the effectiveness and durability of new materials, regenerative therapies, and diagnostic technologies, and the development of international consensus guidelines to harmonize diagnostic and therapeutic approaches, facilitating evidence‐based practice and improved patient outcomes [22, 90].

## 11. Limitations

Although this review synthesizes valuable data on the management of DCLs, the included studies exhibit several methodological limitations. These limitations include small sample sizes, short follow‐up periods, and significant heterogeneity among the trials. Additionally, variability in diagnostic criteria for reversible versus irreversible pulpitis across studies may affect the consistency and generalizability of the findings.

## 12. Conclusions

In conclusion, minimally invasive treatments such as SCR, stepwise excavation, and pulpotomy using bioactive materials like MTA and Biodentine offer effective alternatives to traditional root canal therapy in managing DCLs. Clinicians should consider these biologically driven approaches, particularly in cases where pulp vitality preservation is critical. Proper case selection, accurate diagnosis, and adequate coronal sealing are essential for ensuring the success of these treatments. However, significant gaps remain in the long‐term effectiveness of these approaches, with the need for larger‐scale clinical trials and standardized diagnostic criteria for DCLs. Future research should also explore the use of newer materials, such as A‐PRF+, in pulp‐preserving therapies and assess their long‐term outcomes. Standardizing treatment protocols and follow‐up periods will be essential to improving the consistency and reliability of findings across studies.

**Nomenclature**
AI: Artificial intelligence
A‐PRF+: Advanced platelet‐rich fibrin
CBCT: Cone‐beam computed tomography
CEM: Calcium‐enriched mixture
CH: Calcium hydroxide
DCL: Deep carious lesion
DPC: Direct pulp capping
IL: Interleukin
MTA: Mineral trioxide aggregate
RCT: Root canal treatment
RDT: Remaining/residual dentin thickness
RMGIC: Resin‐modified glass ionomer cement
SCR: Selective caries removal
SRFD: Selective removal to hard dentin
SRSD: Selective removal to soft dentin
TNF‐α: Tumor necrosis factor‐alpha
VPT: Vital pulp therapy.

## Author Contributions

Conceptualization and study validation: Negar Gholizadeh and Nafiseh Zarenejad. Implementation and supervision: Mahdi Gholamrezaei Saravi, Hoorieh Abedi, and Tahereh Molania. Writing and reviewing: Negar Gholizadeh, Hoorieh Abedi, Nafiseh Zarenejad, Mahdi Gholamrezaei Saravi, and Tahereh Molania.

## Funding

The current study received no funding.

## Disclosure

All authors have read and approved the final version of the manuscript.

## Ethics Statement

The authors have nothing to report.

## Consent

The authors have nothing to report.

## Conflicts of Interest

The authors declare no conflicts of interest.

## Data Availability

Data sharing is not applicable to this article as no datasets were generated or analyzed during the current study.
